# Decision-Making Dilemmas within Integrated Care Service Networks: A Systematic Literature Review

**DOI:** 10.5334/ijic.6458

**Published:** 2022-11-17

**Authors:** Jessica Michgelsen, Ludo M. Glimmerveen, Carina A. C. M. Pittens, Mirella M. N. Minkman

**Affiliations:** 1 Athena Institute, Vrije Universiteit Amsterdam, Vilans Centre of Expertise for Long-term Care, The Netherlands; 2 Department of Organisation Sciences, Vrije Universiteit Amsterdam, The Netherlands; 3 Athena Institute, Vrije Universiteit Amsterdam, The Netherlands; 4 TIAS School for Business and Society/Tilburg University, Vilans Centre of Expertise for Long-term Care, The Netherlands

**Keywords:** integrated care, networks, governance, decision-making, dilemmas

## Abstract

**Introduction:**

The diverse nature of people’s care needs requires collaboration between different organisations and sectors. One way of achieving such collaboration is through integrated care service networks. Decision-making is considered an important aspect of network governance and key to achieve further integration of care services. As integrated care scholars only implicitly seem to touch upon the issue of decision-making, we aimed to identify multiple decision-making dilemmas.

**Theory and Methods:**

A systematic literature review was conducted of eighteen empirical studies in which decision-making dilemmas in integrated care service networks were inductively identified. To frame and understand these dilemmas, we partly drew on Provan and Kenis’ governance models and their hypothesised decision-making dilemma for service networks.

**Results:**

Identified decision-making dilemmas included 1) autonomy versus interdependence, 2) diversity versus coherence, and 3) self-interest versus common goals. In line with Provan and Kenis’ hypothesis, we highlight a cross-cutting dilemma of inclusiveness (all viewpoints are considered hence widely supported decisions) vs. efficiency (reaching timely decisions).

**Discussion and conclusion:**

We believe that network- and ‘systemic’ stakeholders both need to reflect upon and learn from decision-making dilemmas to work towards widely supported and adequate decisions. This is important for achieving aligned and holistic care services that many people desire.

## Introduction

Contemporary care provision increasingly requires joint efforts by informal carers, professionals and organisations from various care and life domains. When people simultaneously use services provided by -for example- primary care organisations, hospitals and social care agencies [1], alignment across organisational and sectoral boundaries becomes crucial [2,3]. Creating alignment may, at the professional level, lead to e.g. improved exchange of information, enhanced trust between care providers and better collaboration between professionals [4,5]. At the level of clients and informal carers, cross-organisational alignment holds the potential to better meet clients’ diverse needs as of taking a more holistic approach [6]. One way of achieving such alignment is through the establishment of integrated care service networks, comprising “a group of three or more autonomous organisations working together across structural, temporal and geographic boundaries to implement a shared population health or health services strategy” [7, p. 13]. Participation of different organisations in such networks may thus be beneficial for both professionals as well as clients and their families.

In this literature review, we focus on the dynamics of decision-making in integrated care service networks. Decision-making is considered an important ingredient of effective integrated care network governance and is key to achieve further integration of care services [8]. Provan and Kenis highlight the need for a form of governance in networks that focusses on coordination and control of joint actions across the network [9]. Decisions are argued to precede action(s) as they define “what is intended to be done” [10, p. 114]. At the same time, extant public administration and organisational literature shows that decision-making within networks presents its own dilemmas [11]. For example, network stakeholders usually bring different organisational cultures and frames of reference for dialogues to the table, which can hamper the decision-making process [11,12]. It is also frequently recognised that network stakeholders may have to deal with decisions that are beneficial for the network but are not in line with their organisation’s goals or interests [11]. Although network stakeholders aim for a win-win situation for both the network and each participating organisation, they seem to regularly fail to do so and result in complex interactions that require an extensive amount of time and energy [11].

Failing to address dilemmas in decision-making processes may add to the failure of networks that try to achieve more integration of health and social care services. When decisions are not made or not committed to by those needing to implement them, integrated care service activities can be delayed, discontinued, or even entirely hampered. Accordingly, decision-making dilemmas and possible ways to deal with them in the health and social care setting need to be identified [13]. Little seems explicitly written about arising decision-making dilemmas within the integrated care field. While ample literature focusses on interprofessional and interorganisational care collaborations, integrated care scholars only tend to implicitly touch upon the issue of decision-making [see e.g. 14,15,16]. Consequently, the understanding of decision-making within integrated care service networks seems fragmented. Therefore, we systematically searched for emerging decision-making dilemmas in the context of integrated care. This literature review seeks to answer the following research question: *What empirical evidence is available on dilemmas of decision-making within integrated care service networks?*

## Theory and methods

### Theoretical background

The governance of networks can be conceptualised in numerous ways. One way of considering network governance is through the lens of collaborative governance [17,18]. This involves the engagement of multiple organisations in collective, consensus-oriented, and deliberative decision-making aiming to make or implement public policies and procedures [17]. Others also highlight collaborative negotiations and coordination in e.g. health care as an important element of network governance [16,19]. Another approach is to consider governance more in terms of a (social) structure and the consequences of this structure on network effectiveness [20]. Provan and Kenis [9] take this governance-as-structure-approach in their often-cited article on three different network governance models: 1) participant-governance, 2) lead-organisation governance, and 3) netwerk-administrative-organisation (NAO) governance. In a *participant-governance* model, a large subset of network stakeholders (e.g. care professionals, managers and/or directors) are involved in decision-making. In a *lead-organisation governance* model, one organisation is responsible for key decisions in the network. In a *NAO*-*governance* model, an external organisation or individual -often referred to as facilitative coordinator- is hired for the coordination of the network. In this model, other network stakeholders are still responsible for strategic decisions within the network [9]. Provan and Kenis’ article ended with (amongst others) the following hypotheses:

- *Networks face a tension between the need for administrative efficiency and inclusive decision-making*.
- *Inclusive decision-making will be favoured in shared-governance models*.
- *Efficient decision-making will be favoured in lead-organisation-governance models*.
- *There will be more of a balance between inclusive and efficient decision-making in NAO-governance models, but efficiency will be favoured*.

In order to structure and understand our diverse findings, we partly categorised the studied integrated care networks according to Provan and Kenis’ [9] governance models. While we recognise that Provan and Kenis’ study has been published over a decade ago, empirical studies in the field of integrated care with an explicit focus on decision-making dynamics in differently governed care service networks remain scarce. There is, however, increasing attention for clinical (shared) decision-making between clients and professionals, or different professionals [21]. These ways of decision-making occur at the micro level of integration and may bring along other types of dilemmas (e.g. more related to preferred clinical treatment options among individual clients) when compared to the meso level of interorganisational alignment (e.g. related to financial distributions among parties) [7]. Hence, in this literature review we only focus on meso-level integrated care decision-making dilemmas.

### Methods

#### Systematic search of the literature

A systematic search of peer-reviewed articles was completed in June 2021. The articles were obtained from the electronic databases SCOPUS and Web of Science (step 1). In this search, key terms were based on the following concepts in title and/or abstract: 1) *decision or decision-making*; and 2) *inter-organisational* or *inter-professional* or *governance*; and 3) *network* or *collaboration* or *partnership* or *relationship* or *group*; and 4) *health care* or *social care*. The search syntax can be found in Appendix A. All search results were imported in the literature management system Mendeley and duplicates were removed. Two researchers independently screened the titles and abstracts. Articles were considered eligible for inclusion if they: 1) were published between 2008 and (until June) 2021, which is the time period between the previously described publication of Provan and Kenis [9] and the current study; 2) were published in peer-reviewed journals; 3) were written in English; 4) had an abstract and full-text available; 5) focussed on health care and/or social care; and 6) included empirical data that focussed in some depth on the decision-making at the level of integrated care service networks. Articles were therefore excluded if they focussed on 1) micro-level clinical (professional-client) decision-making; and 2) interprofessional partnerships between care professionals with the goal of making clinical decisions for clients. These in- and exclusion criteria were agreed upon by all authors prior to the screening of titles and abstracts. Two authors independently screened the articles’ titles and abstracts (step 2). Differences in included articles were discussed and resolved by consensus between the two authors. Full-text review was conducted by one author.

#### Data extraction and analysis

First, basic characteristics of the included articles and studied integrated care service networks were extracted by using a deductively developed data sheet. The data sheet captured the articles’ authors, year of publication, country where the study was conducted, sector(s), research methods, network stakeholders’ description and employed governance model of the network(s) (participant-governed, lead-organisation governed, or NAO-governed). The governance models were not explicitly mentioned as being one of those three categories by the original authors. Therefore, governance models were derived based on descriptions in the article text. For instance, a network was identified as employing a participant-governed model if the text described that all/most network stakeholders were involved in e.g. coordination activities and decision-making. Lead-organisation networks were classified as such when described that one organisation was assigned to make key decisions. NAO-governed networks were identified based on descriptions of an external party/person being responsible for e.g. coordination of the network.

Second, following a qualitative analysis approach, each text fragment related to decision-making dynamics and dilemmas were inductively coded by JM [22]. As such, when the word ‘decision’ was mentioned in the text, the described processes around it (e.g. unclear roles or competing agendas) were coded according to the concepts used by the authors to stay as closely as possible to the original text. These coded elements of decision-making dynamics were derived from each original study separately and were added to the data sheet. Third, one author (JM) grouped the codes from each study into categories based on their similarities and differences. From these categories, two authors (JM and LG) inductively generated themes that formed the identified decision-making dilemmas. Fourth, to understand the diverse decision-making dilemmas and partly build on an existing framework, one author (JM) coded text elements that indicated favoured ways of decision-making: a (more dominant) tendency towards inclusiveness or efficiency. These elements were added to the data sheet. If both ways seemed present, we indicated this in the data sheet. Fifth, a first draft of the results based on these decision-making dilemmas was written by one author (JM). All authors commented on this draft through which ‘third order interpretations’ were developed [23]. This led to a new structure of describing the decision-making dilemmas (i.e. dilemmas occurring at different levels within the network(s)). The dilemmas were illustrated using quotes from the original studies.

## Results

### Overview of selected studies

**Figure 1:**
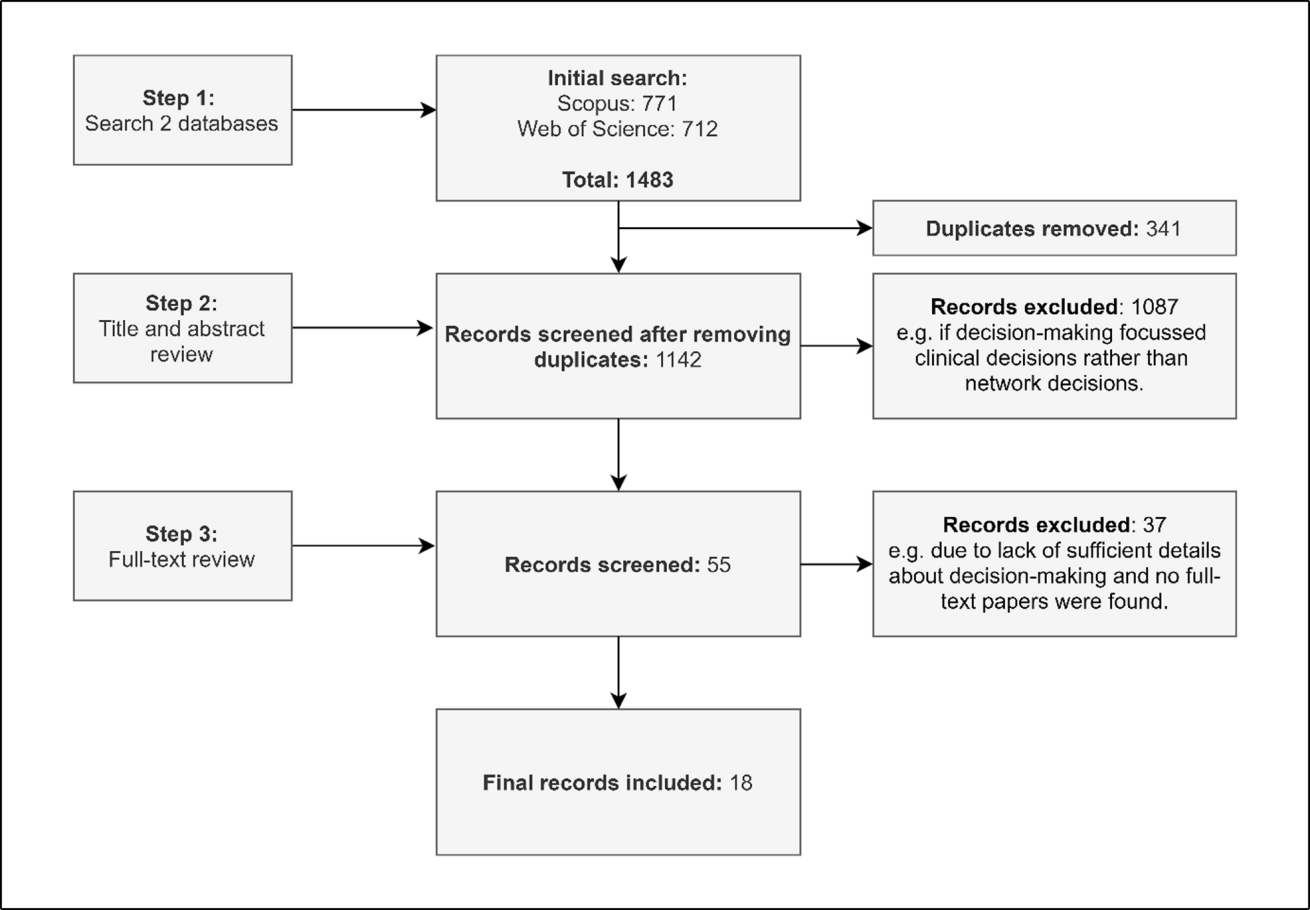
PRISMA flow of article selection process.

The initial search strategy yielded 1483 articles. The article selection process is illustrated in Figure 1. After removing 341 duplicates, we remained with 1142 articles for screening title and abstract. The title and abstract review resulted in the exclusion of 1087 unique articles mainly due to a focus on clinical decisions on the micro-level, leaving 55 articles for full-text reviews. The full-text review resulted in the exclusion of 37 articles for the following reasons: No sufficiently detailed information about decision-making (e.g., who is involved in what type of decisions) (n = 33); no full-text available (n = 3); or because another included article from the same author and project was already included and provided more details about decision-making (n = 1). This led to a final subset of 18 included articles [24,25,26,27,28,29,30,31,32,33,34,35,36,37,38,39,40,41].

Table 1 provides an overview of the 18 included articles in terms of authors, reference number, country and sector, methods, network stakeholders, network size, governance model, favoured decision-making way, and elements of decision-making dilemmas. The articles covered integrated care service networks in the United States [24,25,26,29,34], Afghanistan [27], New Zealand [28], England [30,32,36], Belgium [31], Australia [33,41], Central America [35], Sweden [38], Brazil [39], Chile [37] and Canada [40]. In all studies, except for one [34], some form of qualitative research methods were employed including interviews, focus group discussions (FGDs), observations and document analyses. Six studies also used surveys as an additional data collection method [24,26,27,28,29,34]. Twelve studies covered participant-governed networks and four studies focussed on networks governed by a lead-organisation [28,30,35,39]. One study included a NAO-governed network [37]. The remaining study included 2 participant- and 2 lead-organisation governed networks [25]. Hence, most integrated care service networks were participant-governed.

**Table 1:** Summary of included articles on decision-making in integrated care service networks.


AUTHORS	REFERENCE NUMBER	COUNTRY AND SECTOR	METHODS	NETWORK STAKEHOLDERS	NETWORK SIZE	GOVERNANCE MODEL	FAVOURED DECISION-MAKING WAY: INCLUSIVE OR EFFICIENT?	ELEMENTS OF DECISION-MAKING DILEMMAS

Ales et al. (2011)	24	United States of America, Health care	Survey and interviews	Collaboration between university departments (n = 3) and (medical) education organisations (n = 6)	9 organisations	Participant-governed	Inclusiveness in terms of key decisions (mission, governance, financial deviations from budget), otherwise efficiency	1. Loss of autonomy vs. shared commitment 2. Finding appropriate group size 3. Flexibility vs. structure

Alexander et al. (2010)	25	United States of America, Health care	Multiple case study (interviews)	4 different networks consisting of health care providers, purchasers and clients	Unspecified	2 participant-governed and 2 lead-organisation	Described dilemma of inclusiveness (consensus based) vs. efficiency (exclusive, smaller decision-making groups)	1. Competing agendas stakeholders 2. Negative historical relationships 3. Balancing long-term goals versus specific goals 4. Culture differences (diversity)

Alidina et al. (2016)	26	United States of America, Social care	Survey and interviews	Network of patient-centred medical homes with physicians, managers, staff	13 organisations	Participant-governed	Unspecified	1. Decision-making costs 2. Time needed to build relationships 3. Communication barriers

Anwari et al. (2015)	27	Afghanistan, Health care	Case study (survey, system data, focus group discussions)	Provincial public health coordination committee including 21 members	10 organisations	Participant-governed	Efficiency (several subcommittees were established for e.g. decision-making. Information was shared top-down)	1. Lack of commitment to the network and decisions 2. No transparent decision-making

Barnett et al. (2009)	28	New Zealand, Health care	Survey and interviews	21 different district health boards. Chairs appointed by Minister of Health	11 members per board	Lead-organisation governed	Inclusiveness (striving for non-mandated horizontal relationships)	1. Unclear roles 2. Power imbalances 3. Dual accountabilities

Carstens et al. (2009)	29	United States of America, Social care	Survey and interviews	13 networks with organisations in child protection services, mental health, drug and alcohol, human services	29 organisations	Participant-governed	Inclusiveness in the design (18 types of stakeholders were involved in decisions), but for efficiency purposes families were brought (too) late to the table	1. Competing agendas stakeholders 2. Knowledge differences 3. Lack of cooperation 4. Autonomous stances 5. Silo thinking

Checkland et al. (2013)	30	England, Health care	Multiple case study (surveys, observations, interviews)	8 clinical commissioning groups (GPs, lay members, managers, nurses, local authority, others)	Unspecified	Lead-organisation governed	Efficiency (most groups chose to split up in subcommittees for efficiency purposes)	1. Unclear external accountability to NHS boards constrain rapid decisions 2. Smaller groups for efficiency vs. opened membership for inclusion

De Regge et al. (2018)	31	Belgium, Health care	Focus group discussions	Hospital stakeholders network with physicians, administrators and clients	Unspecified	Participant-governed	Inclusiveness (decisions must consider each point of view and include all voices)	1. Competing agendas: organisation vs network 2. Differences in perceptions

Gale et al. (2017)	32	England, Health care	Interviews	Health research network (nurses, pharmacists, health trainers, clients)	Unspecified	Participant-governed	Inclusiveness (respecting and including cultural wisdom of all parties involved)	1. Lack of trust and relationships 2. Set rules and procedures vs. adaptability 3. Recognising peoples’ expertise

Harris et al. (2017)	33	Australia, Health care	Case study (interviews, workshops, document analysis)	Network of 6 hospitals, rehabilitations services, mental health and community health services and residential aged care services.	22 committees, 9 approved purchasing units, 1 steering committee, 6 hospitals and a number of unspecified other organisations	Participant-governed	Inclusiveness within committees (deliberative processes), and efficiency due to establishing ‘higher-level’ and ‘lower-level’ committees	1. No formal decision-making process 2. Lack of evidence for decisions 3. Lack of coordination, communication, collaboration 4. Not wanting to include outsiders

Hearld et al. (2012)	34	United States, Health care	Quantitative surveys	14 health care alliances (insurer companies, employers, care providers, government organisations, consumer organisations, others)	1191 members from various organisations (570 filled in the survey)	Participant-governed	Inclusiveness (open and inclusive alliance decision-making processes)	1. Information asymmetry 2. Not devoting enough time to network activities

Hoey, Pelletier (2011)	35	Central America, Health care	Interviews	Network of Ministry of Health, United Nations, NGOs	3 Ministry of Health actors, 1 United Nations member, 4 NGO actors	Lead-organisation governed	Efficiency (perspectives of NGO-employees are not considered by the MoH when taking decisions)	1. Conflicting views 2. Power imbalances 3. Not wanting to include outsiders 4. Mistrust

Marshall et al. (2021)	36	England, Health and social care	Interviews	Staff and managers from different care homes, GPs, social workers, hospices, local authorities (bottom-up networks created during COVID-19)	Unspecified	Participant-governed	Efficiency (due to rapid decisions needed during COVID-19)	1. Autonomy vs. interdependence 2. Self-interest vs. common goals 3. Different interpretations of decisions

Montenegro & Mercado (2019)	37	Chile, (Mental) health care	Interviews and participant observations	Local community mental health service network including service users/community members and mental health professionals	Unspecified	Network-administrative governed	Inclusiveness (the network has to be big because big decisions are made, it needs to be representable)	1. Diversity vs. coherence 2. Self-interest vs. common goals 3. Representation vs. mandate 4. Inclusiveness vs. efficiency

Ramgard, Forsgren, Avery (2017)	38	Sweden, Health and social care	Dialogue, reflections, research circles, workshops, focus group discussions	Voluntary network representing 33 municipalities	>50 members	Participant-governed	Efficiency (decisions were mainly top-down made by politicians)	1. Competing agendas organisations vs. network 2. Power imbalances

Santos, Giovanella. (2014)	39	Brazil, Health care	Case study (interviews, focus group discussions, observations, document analysis)	Regionalised network representing 19 municipalities (state secretary of health, administrators, managers, health professionals)	Unspecified	Lead-organisation governed	Efficiency (politicians formulated the agendas leaving insufficient space for dialogue between stakeholders)	1. Power imbalances 2. No problem-solving strategies 3. Lack of autonomy

Valaitis et al. (2018)	40	Canada, Health care	Interviews	Primary care and public health collaboration (health care professionals, managers, policy makers, researchers, consultants, coordinators, health educators)	>70 members	Participant-governed	Inclusiveness (striving for shared decision-making across disciplines)	1. Unclear roles 2. Culture differences (diversity) 3. Lack of common language

Walker, Smith, Adam (2009)	41	Australia, Health and social care	Interviews	Primary care partnership committees (CEOs, managers, care service providers)	31 committees	Participant-governed	Inclusiveness (transcend differences and find common ground to achieve shared goals)	1. Breaches of trust 2. Competing agendas organisations vs. network


### Three dilemmas in decision-making processes

**Figure 2:**
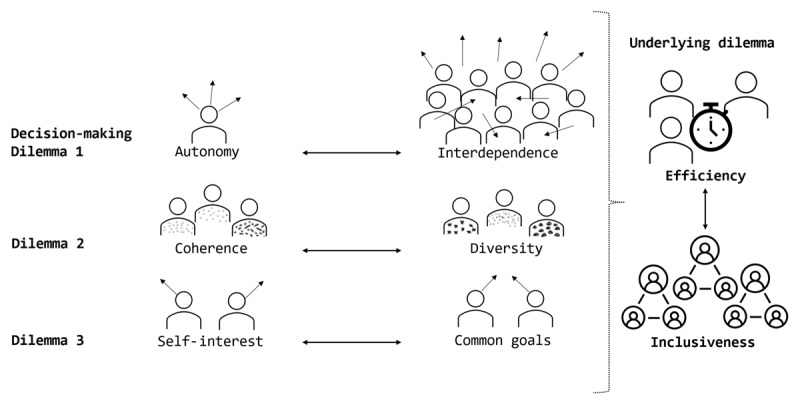
Illustrated decision-making dilemmas within integrated care service networks.

The following paragraphs outline three dilemmas for decision-making within integrated care service networks that are illustrated in Figure 2. We first shortly describe the identified dilemma(s) and in which network-governance models they emerged. As a second part, we seek a more in-depth analysis by describing elements of the dilemmas that seem to occur on different levels. Finally, we discuss how these three dilemmas seem underpinned by a cross-cutting dilemma of finding a balance between inclusiveness and efficiency within decision-making processes.

#### Dilemma 1: Autonomy versus interdependence

As identified in eight articles, one of the dilemmas in decision-making processes concerns the network stakeholders’ sense of autonomy while, at the same time, being dependent on each other in their pursuit of integrating services [24,29,31,32,33,36,40,41]. On the one hand, some network stakeholders are used to make decisions in an individual manner and may not be able to give up (some of) their autonomy. On the other hand, working in networks implies that stakeholders are at least to some extent interdependent. This interdependence often requires, to a certain degree, some form of collaborative decision-making. The eight articles in which this dilemma became apparent all concerned participant-governed networks with stakeholders such as care practitioners and managers striving for consensus.

This dilemma seemed to appear mainly at two levels: (1) operational decisions related to integrated services, and (2) strategic decisions mainly concerning the position of each partner organisation. When it comes to the first level, care practitioners are sometimes seen as autonomous professionals that are not used to making collaborative (operational) decisions: “Practitioners accustomed to working alone and unwilling to work within a team environment can present a significant barrier to public health and primary care collaboration” [40, p. 396]. Others stated that “it’s very hard to get a clinician to say they will stop doing something”, indicating a sense of unwillingness to let other network stakeholders co-decide on one’s activities [33, p. 13].

These autonomous attitudes are, however, part of the reality when practitioners from different disciplines strive for collaboration: “Collaboration in health care is based on the premise that professionals want to work together to provide better care…At the same time…they have their own interests and desire to retain a degree of autonomy and independence” [31, p. 2]. It also requires the recognition of practitioners’ professional expertise and jurisdiction over their practices and putting in the needed time to build trust [32]. Thus, autonomous behaviour by care practitioners is a legitimate reality of (integrated) care service delivery due to their professional expertise but can also form a barrier to collaborative decision-making.

When it comes to the second level, managers and leaders sometimes seem to take autonomous stances in strategic decision-making processes due to the position of their own organisation. As described: “each partnering organisation must be willing to accept the reality that there will be… a potential loss of autonomy and prestige in an area that it had previously considered itself to be a leader” [24, p. 15]. Autonomous stances may come from the notion that “change [due to collaborating in networks]… may entail modifications to how an organisation functions, its purpose and identity in the community, or perhaps even its survival” [34, p. 275]. Such notions can hinder the network’s collaborative strategic decision-making [29, p. 351]. Hence, we foresee a dilemma between maintaining the organisation’s autonomy whilst being dependent on each other’s organisations to achieve integrated care.

#### Dilemma 2: Diversity versus coherence

According to nine articles, another dilemma includes the search for coherence whilst facing a diversity between network stakeholders [25,28,29,34,35,37,38,39,40]. On the one hand, it seems easier to make decisions in a more coherent group as stakeholders try to achieve similar goals by finding common ground and shared understanding [34]. On the other hand, integrated care networks often emerge as of the need to collaborate between a diverse set of disciplines and organisations. Such a diverse group may hamper decision-making due to differences in viewpoints, communication styles, historical relationships, and/or power. Four of these networks were participant-governed [29,34,38,40], three were governed by a lead-organisation [28,35,39], one NAO-governed network [37], and one study covered a mix of 2 participant- and 2 lead-organisation-governed networks [25].

This dilemma seems to appear at similar levels as mentioned in dilemma 1: (1) operational decisions related to integrated care services, and (2) strategic decisions in which power imbalances come to play a role. At the operational level, decision-making challenges mainly arose as of diversity in viewpoints, communication styles and/or historical relationships between different care practitioners. In terms of diversity in viewpoints, it was for instance mentioned that service providers did not understand the added value of the proposed integrated care service intervention. While these benefits were clear for the clients themselves [29]. Others noted that a diversity in viewpoints is particularly evident between professionals operating at different levels within the organisation (e.g. managers vs. care providers) [31].

Decision-making processes were also hampered by different communication styles and the interpretation of words by different people. It was stated in a participant-governed public health and primary care network that: “The words are a problem. They have been and they continue to be. They are almost interchangeable, but they mean different things to different people” [40, p. 384].

In relation to diverse historical relationships, it was mentioned that “…personal relationships [among network stakeholders] was a facilitating factor…in reaching early consensus…” [25, p. 656]. However, negative shared experiences from the past may forestall reaching a (final) decision because of continuing disagreements. A mix between existing positive historical relationships (related to the coherence side of this dilemma) and creating new ones (related to diversity) seems therefore desired [25].

At the second level, when it comes to decision-making at the strategic level of the network, differences in power seem to be the main underlying challenge [25,28,29,31,35,38,39]. An example: “The low autonomy of health administrators compared to In this network, political stakeholders formulated the agendas, reduced roundtable discussions, and left insufficient space for dialogue with health administrators. This demonstrates how dominant network stakeholders excluded less powerful stakeholders from decisions about new health policies. the municipal executive power also represented a barrier to strengthen collective decisions” [39, p. 626]. Thus, reducing the diversity of perspectives may smoothen the process of reaching a decision as this is easier with likeminded, but it can also hamper the support for the decision and thereby peoples’ willingness to act upon it. Hence the need to involve a diverse set of stakeholders, even though this may be challenging.

#### Dilemma 3: Self-interest versus common goals

The third identified dilemma involves the self-interests of stakeholders and/or their own organisations versus the common goals of the network, as illustrated in twelve articles [24,25,28,29,31,33,34,36,37,38,39,41]. On the one hand, stakeholders may strive for their own organisation’s goals when making decisions in a network. This is a consequence of the notion that they cannot fully ‘separate’ themselves from their affiliations with their organisations. On the other hand, organisational goals may not always be aligned with the network’s goals which is likely to hamper collaborative decision-making. This dilemma occurred in seven articles with participant-governed networks [24,29,31,33,34,36,38,41], in two lead-organisation networks [28,39], one NAO-governed network [37], and one article focussed on 2 participant- and 2 lead-organisation governed networks [25].

This dilemma mainly emerged at two levels which are different from the previous dilemmas: differences in interests between (1) network stakeholders from various organisations, and (2) network stakeholders from the same organisation. At the first level, Walker et al. [40] noted that there is always a chance that network stakeholders pursue organisational interests rather than network interests. Others pointed out: “A challenge…is sorting out divergent interests without putting the enterprise at risk of dissolving” [25, p. 659]. “If the network was merely a collection of self-interested parties, reaching consensus was a complex and slow task” [37, p. 6]. Ales et al. took a different approach and highlighted that the self-interests of each partner should not be threatened by ensuring the collaborative’s goals to be aligned with the organisational goals.

Such competing interests do not only occur between network stakeholders from different organisations but also between stakeholders working within the same organisation. As illustrated in the following quote: “What the hospital is concerned about: finances, organisational capacity and risk management” versus “what the clinician is concerned about: patients” [40, p. 15]. Or in another network, it was noted that striving for consensus delayed the network’s progress of a key initiative: “…We don’t have consensus… the doctors are over here, and the purchasers are over here…” [25, p. 651]. Naturally, not only stakeholders from different organisations but also those from the same organisation can have diverging interests. Finding a balance between individual interests at various levels and overall network goals thus seems a challenging necessity for decision-making.

#### Underlying decision-making dilemma: inclusiveness vs. efficiency

In line with Provan and Kenis’ hypotheses, we highlight a dilemma around decision-making inclusiveness versus efficiency. The aforementioned dilemmas share a commonality in the sense that they all relate to the question of whom (with which interests) to include in what decision? Alexander et al. specifically highlighted this: “Often alliances are faced with a choice of establishing a smaller executive committee to make key decisions [efficiency] versus taking a more consensus-based approach of involving the entire alliance in virtually all decisions [inclusiveness]” [25, p. 652]. Establishing smaller (sub)groups in a network then implies a more efficient decision-making process due to fewer chances of autonomous behaviour (dilemma 1), fewer diverse perspectives (dilemma 2) or competing interests (dilemma 3). In this sense it seems that efficiency can be enhanced by making timely decisions with a small group of aligned stakeholders. In six articles we were able to identify a tendency towards efficient decision-making ways, of which three employed a participant-governed network model [27,36,38] and three a lead-organisation model [30,35,39].

This search for efficiency, however, seems to contradict the wish in (especially participant-governed) networks to include all (relevant) network stakeholders in decision-making processes. If everyone is included, chances are highest to reach widely supported, adequate, and committed to decisions. For this to happen it seems necessary to include those interdependent on each other (dilemma 1), with a diverse set of views from different disciplines and organisations (dilemma 2), who can represent various interests (dilemma 3). The disadvantage of such an inclusive approach is that it “necessitates much greater commitment of time and process-oriented activities. This in turn may lead to delays [in decision-making and therefore care integration] and possible burnout [in network stakeholders]” [25, p. 652]. In 9 articles we were able to identify a tendency towards inclusive decision-making ways, of which 8 employed a participant-governed network model [24,28,29,31,32,34,40,41] and 1 NAO-model [37].

Thus, there seems an inevitable trade-off between wanting to include all relevant network stakeholders in decision-making for the sake of widely supported and committed to decisions (inclusiveness), whilst being able to reach decisions in a considerable amount of time (efficiency).

## Discussion

In this literature review, three main patterns of decision-making dilemmas were identified within a diverse range of integrated care service networks across the world. In line with Provan and Kenis’ [9] hypotheses, we also highlight a cross-cutting dilemma of inclusiveness vs. efficiency. Decision-making inclusiveness is required to reach widely supported and adequate decisions that are built on a broad range of perspectives. This seemed particularly favoured by stakeholders in participant-governed networks looking for consensus. At the same time, some of these participant-governed network stakeholders strived for an efficient decision-making process to reach decisions in a timely fashion. In some of these cases, efficient decision-making then implied the deliberate exclusion of stakeholders with opposite views or interests. These findings highlight that a sole focus on inclusive designs will not meet the total demands of dealing with the complexity of an integrated care network [42,43]. Even in consensus-oriented participant-governed networks, stakeholders still faced the need to balance inclusion with efficiency.

Accordingly, the first contribution of our literature review is that participant-governed networks’ emphasis on inclusive decision-making designs may be less self-evident than hypothesised by Provan and Kenis [9]. Even though it was common in most participant-governed networks to strive for consensus, the actual (described) practices of decision-making often turned out to be minority groups making decisions. Or when it comes to network membership, some networks explicitly did not involve certain important stakeholders. This was for instance the case when autonomous behaviour or self-interests played a role in decision-making dynamics. In these cases, there seemed to be a tension between the goals and interests from the individual network stakeholders on the one hand, and the network as a whole on the other hand. This points out an important implication for the collaborative governance perspective in which inclusive, collective, consensus-oriented collaborations are typically strived for [17,18,44]. However, in practice, we see that decision-making behaviour in networks can often be unstructured, nonlinear and unpredictable [45]. The proposition that collective and integrative processes will lead to better decisions, and therefore better policies, procedures and health outcomes [44] then also becomes questionable. Thus, with the inevitable existence of contradicting interests and goals from different people from different organisations (otherwise it would not be an interorganisational network), true collaborative decision-making processes may possibly not always be as feasible and/or effective as often longed for in integrated care [46].

The second contribution of our literature review stems from our specific focus on integrated care practices. While Provan and Kenis, amongst many other authors [e.g. 47], focus on the broader public sector, we give an overview of dilemmas in health and social care systems across the world. These health and social care systems bring along specific dynamics in terms of opportunities and limitations in interorganisational collaborations with unequal partners. Or as Meurs recently put it in the example of the Dutch health care system: “It is often said in health care that there are too many subdivisions in the system. That these subdivisions are the root cause of poor care. This suggests that the essential act of providing care can be limited by constructed boundaries through policies, rules and regulatory mechanisms” [48, translated by the current authors from p. 9]. Others highlight a similar idea that care integration is not merely dependent on ‘systemic alignment’, but also relies on the efforts of care professionals and other network stakeholders themselves to overcome bureaucratic or other systemic barriers. They therefore suggest to actively look for integration opportunities within the system while explicitly navigating and addressing its contradictory features [49]. Thus, we want to acknowledge how tensions and dilemmas often cannot be ‘solved’ or erased in the system as each system inherently has its own limits. That means that care professionals and other network stakeholders will always face the need to constructively deal with emerging dilemmas when they work across the boundaries of their professions, organisations and care domains.

While we underline the importance of actively navigating dilemmas entrenched in care systems, we also acknowledge the importance of bringing about systemic change that favours service integration. We argue that many of the decision-making dilemmas are likely to stem from financial, accountability and (other) power mechanisms in the wider health and care systems in which individual networks are embedded. While the care systems in the countries included in this literature review are diverse, health and social care professionals across these countries face similar underlying tensions that may originate from wider system contexts.

An example of such a ‘systemic’ tension is the uneven distribution of accountability and responsibilities over different governance levels. This can be illustrated by looking at one of the studies in this review in England [30]. In 2010, major policy reforms occurred whereby various initiatives had been put into place to integrate care services at the local level [50]. One of the initiatives was the creation of primary care (network-based) Clinical Commissioning Groups (CCGs) that were held responsible for overseeing local market mechanisms [50,51]. These CCGs were, however, organised with a bottom-up approach which essentially meant a different structure for each site. On top of that, multiple sub-committees such as executive committees, audit committees, remuneration committees et cetera were established [52]. McDermott et al. [52] therefore concluded that in the context of these complex structures, decision-making responsibilities and authorities were unclear and did not seem to bring general practitioners any closer to the decision-making process (as was intended). The past few years, the government shifted responsibilities and accountability mechanisms even more to the local levels which adds to the blurry lines of decision-making responsibilities. To conclude, the above illustrates how changing environments can influence decision-making in networks and seems to require a great sense of adaptability, flexibility and alertness among network stakeholders [51].

Another structural tension that is argued to influence decision-making dynamics is the focus on competition instead of collaboration; mainly seen as a result of financial mechanisms. For instance, in the context of the United States (in which 5 out of the 16 included studies took place), payment mechanisms usually focus on single organisations working on specific diseases in a particular sector [53]. This contradicts integrated care service networks trying to achieve population-based quality of care by crossing primary, secondary and social care boundaries. It is therefore considered beneficial to incentivise the integration of care services through payment mechanisms that transcend organisational and sectoral boundaries, such as pooled health and care budgets, shared shavings, and financial rewards for improved quality of life [53]. Thus, there seems to be a systematic tension between national governance mechanisms that focus on individual organisations and integrated care service networks that try to overcome this thinking and acting in silos. As long as governance arrangements in health and social care systems are not sufficiently focussed on financially incentivising collaborations, integration of care services inherently remains impeded.

### Practical implications

While the integrated care service networks studied in this literature review are embedded within different care system contexts and involve a great variety of structures and people, the identified decision-making dilemmas seem to transcend these differences. Nevertheless, ways of dealing with these dilemmas will be context specific. When doing so, it is important for care professionals to sometimes go beyond the set-out boundaries of their profession, and to work *with* their differences, sense of autonomy, and diverse interests instead of working *against* these issues or ignoring them. For network stakeholders to figure out how to work with these issues, it might be helpful to explicitly reflect on experienced tensions and dilemmas [54,55,56]. When reflective moments are built in within the network stakeholders’ way of collaborating, it may become easier to deal with the emerging dilemmas over time. It could especially be helpful to exchange these reflections with ‘system’ parties, such as health insurers and government bodies, if faced with organisational or systemic boundaries. In this way, reflections and learnings do not stay within local (network) contexts but can be addressed at regional or national levels [55]. Thus, our main practical recommendation for local network stakeholders is to know when to involve stakeholders from system parties that may be able to influence, address and learn from the occurring dilemmas.

### Study limitations

When drawing conclusions from this literature review, several limitations must be considered. First, not all relevant articles from the examined databases may have been included due to difficulties around formulating inclusive search terms. In most health care literature, decision-making processes refer to clinical professional-client decision-making rather than our focus on decision-making in networks. We tried to address this by employing frequently used search terms in other fields (outside health care). Second, the overall number of included networks found was limited; only one NAO-governed network, six lead-organisation-governed networks and fourteen participant-governed networks. Perhaps this is the result of an earlier made notion that most network stakeholders seem to strive for inclusive designs such as participants-governed networks (which – as the analysis showed – does not mean that decision-making practices are as inclusive as intended). Third, none of the included articles primarily focussed on decision-making. All publications had had a broader scope and included other elements of interorganisational collaboration. Hence, dilemmas in decision-making were often not explicitly stated in the text. Fourth, the chosen methodology of a literature review gives an overview as we aimed for, but does not fully provide in-depth and context specific insights into decision-making. This provides opportunities for further research in integrated care.

### Future research opportunities

The fact that none of the included articles solely focussed on decision-making within integrated care service networks presents future research opportunities. It would be interesting to conduct qualitative studies to gain more in-depth understanding about this underlying trade-off between inclusive and efficient decision-making in care service networks. Another option would be to provide more insights into the steps taken to deal with arising dilemmas and how this can help achieve the actual integration of care services. This may be done via a more action-oriented study towards successful strategies. Finally, the relation between, on the one hand, the process of decision-making and, on the other hand, the outcomes and follow-up of decisions is an interesting subject of study, as it allows us to explore how decision-making can contribute to better care and support for people.

## Conclusion

There is an increasing need to align care services of providers from different organisations, domains and/or sectors. Establishing such alignment often involves long-term processes. It takes time to cultivate collaborations in which diverging interests, goals, roles, interdependencies, commitment, power levels, and relationships are sorted out. These joint efforts call for active consideration of which forms of governance are effective or suitable. Decision-making is an important aspect of governance but receives little explicit attention in the integrated care literature. In this literature review we shed light upon decision-making dilemmas that arise within complex, long-term processes to integrate (more) care services. We believe that dilemmas need to be explicated and reflected upon –by network stakeholders themselves, but also by engaging ‘systemic’ actors in a network’s wider context– to work towards widely supported, adequate, and committed to decisions. This is an important element of achieving aligned and holistic care services that many people need and desire.

## Appendix A

**Search syntax**

(“Decision making” OR “decision-making” OR “decision” AND NOT “shared-decision”) AND (“interorganisational” OR “interorganizational” OR “inter-organisational” OR “inter-organizational” OR “interprofessional” OR “inter-professional” OR “governance”) AND (“network” OR “collaboration” OR “partnership” OR “relationship” OR “group”) AND (“healthcare” OR “health care” OR “health-care” OR “social care”).
